# News and Notes

**Published:** 1903-02

**Authors:** 


### *NEWS AND NOTES.*

(We are indebted to the *Medical Age* for many of these notes.)

---

Dr. J. N. Brawner has been elected a member of the Board of Health.

---

Dr. T. N. Brewster has been reelected Superintendent of the Grady Hospital.

---

A bill is now pending in Congress providing for fifty women nurses in the navy.

---

Three cases of bubonic plague are now under treatment in the Swinburne Island Quarantine Hospital in Lower New York Bay.

---

The American Association for the Cure of Inebriety held its thirty-second annual meeting in Boston, Mass., December 18, 1902.

---

The House of Representatives has been asked for an appropriation of $75,000 for the construction of an army general hospital in Washington.

---

The surgeon-general of the U. S. P. H. and H. M. service will soon call a conference of all the State boards of health to confer in regard to the plague.

---

The Georgia Medical Society, at its annual meeting held at Savannah on January 6th, elected the following officers: President, Dr. J. G. Jarrell; Vice-President, Dr. H. H. Martin; Recording Secretary, Dr. J. O. Cook; Corresponding Secretary, Dr.

H. W. Hesse; Treasurer, Dr. C. B. Lanneau, and Librarian, Dr. J. G. Van Marter.

---

THE Secretary of the Navy in his recent annual report to Congress recommends a permanent ambulance or hospital ship designed for hospital service only, and having a right to fly the flag of the Geneva Conference.

---

THE government physician at the Sac and Fox Indian agency in Oklahoma announces that a large majority of the tribe are afflicted with incurable diseases, and in all probability they will be extinct within the next few years.

---

THE new Register of the Johns Hopkins University shows that there are sixty-nine professors and instructors in the medical department. Two hundred and fifty-eight medical students and seventy-two physicians are attending special courses.

---

ACCORDING to *American Medicine* a sanatorium for the treatment of pulmonary tuberculosis, located at Luray, Va., has the system of ventilation so arranged that all the air for the institution is supplied from the underground caverns. It is claimed that the air of limestone caverns has all the characteristics of the pure rarefied air of high altitudes, and that an investigation of the air in caverns supplying the sanatorium showed it to be of uniform temperature, remarkably pure and free from all germs and dust particles, and especially adapted for the treatment of pulmonary complaints.

---

AN official crusade is about to be instituted in France against the sale of various poisonous essential oils, especially those used in the manufacture of liqueurs and cordials. The Prime Minister, M. Combes, himself a physician, some time ago, according to the

*Medical Record*, officially asked the Academy of Medicine to formulate a list of poisonous essences contained in liqueurs and cordials, intending when the list is received to submit a bill to the Chamber of Deputies restricting the consumption of these compounds, which, he says, are threatening the vitality of the French race. Liqueurs such as absinthe, chartreuse, and the like, are principally aimed at. M. Laborde, who is the chairman of the committee appointed by the academy to comply with M. Combes's request, favors the absolute prohibition of the sale of all the poisonous essential oils except by druggists on physicians' prescriptions. The leading distillers are said to have raised a fund of $200,000 to avert the contemplated legislation.

---

THE New Orleans meeting of the American Medical Association will take place on May 5th to 8th. The Southern Railway has announced a reduced rate of one fare for the round trip from Washington, or from any point on their system to New Orleans and return. Tickets will be on sale May 1st to 4th and will be good for continuous passage in each direction with a final limit of ten days from the date of sale. Tickets can be extended for a longer period, however, provided they are deposited in person by the original purchaser, with the special agent at New Orleans not later than May 12th, 1903, and fee of fifty cents is paid at the time of deposit, when the final limit will be extended to a date not later than May 30th. Further information may be obtained from the Chairman of the Committee on Tranportation, Dr. H. L. E. Johnson, Jefferson Place, Washington, D.C.

